# COX deficiency and leukoencephalopathy due to a novel homozygous *APOPT1/COA8* mutation

**DOI:** 10.1212/NXG.0000000000000464

**Published:** 2020-06-16

**Authors:** Carola Hedberg-Oldfors, Niklas Darin, Christer Thomsen, Christopher Lindberg, Anders Oldfors

**Affiliations:** From the Department of Pathology and Genetics (C.H.-O., C.T., A.O.) and Department of Pediatrics (N.D.), Institute of Clinical Sciences, Sahlgrenska Academy at University of Gothenburg; and Department of Neurology (C.L.), Neuromuscular Centre, Sahlgrenska University Hospital, Gothenburg, Sweden.

## Abstract

**Objective:**

To describe the long-term follow-up and pathogenesis in a child with leukoencephalopathy and cytochrome c oxidase (COX) deficiency due to a novel homozygous nonsense mutation in *APOPT1/COA8*.

**Methods:**

The patient was clinically investigated at 3, 5, 9, and 25 years of age. Brain MRI, repeat muscle biopsies with biochemical, morphologic, and protein expression analyses were performed, and whole-genome sequencing was used for genetic analysis.

**Results:**

Clinical investigation revealed dysarthria, dysphagia, and muscle weakness following pneumonia at age 3 years. There was clinical regression leading to severe loss of ambulation, speech, swallowing, hearing, and vision. The clinical course stabilized after 2.5 years and improved over time. The MRI pattern in the patient demonstrated cavitating leukoencephalopathy, and muscle mitochondrial investigations showed COX deficiency with loss of complex IV subunits and ultrastructural abnormalities. Genetic analysis revealed a novel homozygous mutation in the *APOPT1/COA8* gene, c.310T>C; p.(Gln104*).

**Conclusions:**

We describe a novel nonsense mutation in *APOPT1/COA8* and provide additional experimental evidence for a COX assembly defect in human muscle causing the complex IV deficiency. The long-term outcome of the disease seems in general to be favorable, and the characteristic MRI pattern with cavitating leukoencephalopathy in combination with COX deficiency should prompt for testing of the *APOPT1/COA8* gene.

Mitochondrial encephalomyopathies with cytochrome c oxidase (COX, complex IV of the respiratory chain) deficiency (MIM 220110) is a heterogeneous group of disorders associated with mutations in the mitochondrial genome as well as in nuclear DNA.^1^ Mutations have been identified in genes encoding the 14 subunits of COX (3 encoded by the mitochondrial DNA and 11 encoded by the nuclear genome) and in assembly factors encoded by the nuclear genome.

Loss of function mutations in *APOPT1/COA8* (hereafter *COA8*) encoding cytochrome c oxidase assembly factor 8 have been associated with cavitating leukoencephalopathy with COX deficiency in 7 reported individuals.^2,3^ The disease was characterized by onset in childhood or adolescence of a leukoencephalopathy with cystic lesions predominantly in the posterior part of the cerebrum and sparing of the infratentorial parts of the brain. The presentation has been variable with an acute encephalopathy in early childhood or subtle neurologic signs in adolescence. After the initial symptoms, a period of regression followed with additional disease manifestations, including spastic paraparesis. This period lasted in most cases from a few months up to 2 years, where after stabilization or improvement was reported. One child died 6 months after disease onset because of aspiration pneumonia.^3^

In this study, we describe a 25-year-old woman presenting in late infancy with leukoencephalopathy, COX deficiency, and loss of complex IV subunits associated with a homozygous nonsense mutation in *COA8*. Follow-up including repeat muscle biopsy and clinical investigations revealed a period with regression, followed by stabilization and partial improvement over the years. By protein expression analyses, we provide evidence for a COX assembly defect underlying the complex IV deficiency in human muscle.

## Methods

### Standard protocol approvals, registrations, and patient consents

The study complied with the Declaration of Helsinki, and informed consent was obtained from the patient.

### Morphologic analysis

Open skeletal muscle biopsies from the left vastus lateralis muscle were performed at age 3 years and 9 years in individual II:1. Specimens were snap-frozen in liquid propane chilled by liquid nitrogen for cryostat sectioning and histochemistry. Standard techniques were used for enzyme histochemistry.^4^ For immunohistochemistry, sections were fixed in 4% formaldehyde at 4°C for 10 minutes, washed in TBS-T for 10 minutes, permeabilized in a graded methanol series, washed in TBS-T for 5 minutes, and further processed in a Dako Autostainer using the Dako EnVision FLEX High pH kit. Primary antibodies were applied for 1 hour.

### Western blot

Protein was extracted using SDS-urea buffer at 37°C for 10 minutes and samples cleared by centrifugation (14,000*g*, 5 minutes), and 10 μg was loaded per well on NuPAGE 4%‒12% Bis-Tris gels (Thermo Scientific, Waltham, MA), followed by transfer to polyvinylidene fluoride membranes and subsequent Coomassie staining (gels). Blots were incubated with primary antibodies to complexes I‒V overnight at 4°C and visualized with horseradish peroxidase–conjugated secondary antibodies and SuperSignal West Femto substrate (Thermo Scientific).

### Molecular genetic analysis

Whole-genome sequencing (WGS) was performed on genomic DNA using the TruSeq PCR-free library preparation kit, and the HiSeq X platform was used for sequencing (Illumina, San Diego, CA). The paired-end reads were aligned to the reference genome (hg19) using the CLC Biomedical Genomics workbench (Qiagen, Valencia, CA). Data were analyzed using Ingenuity Variant Analysis (ingenuity.com/products/variant-analysis) (Qiagen). A search for potential compound heterozygous or homozygous variants in candidate genes associated with mitochondrial myopathy that were predicted to be damaging using SIFT and PolyPhen2, affecting a conserved amino acid and not common in the human population, was performed. Confirmation of the variant and analysis of parental samples was undertaken by Sanger sequencing. For functional analysis of the homozygous nonsense mutation identified in *COA8*, total RNA was isolated from frozen skeletal muscle using the RNeasy Fibrous Tissue Mini Kit (Qiagen). RNA was reverse transcribed with the QuantiTect reverse transcription kit (Qiagen), and COA8 complementary DNA was analyzed by PCR with the following primers: forward 5′-GCGGGGAAGAAGACCTTTC-3′ and reverse 5′-AGGCCCAGGCCTTTAGTTT-3′.

### Data availability

The data and detailed protocols are available on request.

## Results

### Case description

This girl was born to healthy unrelated Swedish parents and has a healthy sister. She was born at term after a normal pregnancy. Birth weight was 3,580 g, and length was 50 cm. The perinatal period was normal except for dislocated hips that were treated with von Rosen splint. Early psychomotor development and growth were normal. At age 2 11/12 years, she deteriorated in association with pneumonia and acute otitis media with progressive development over the following months of fatigue, dysarthria with loss of words, dysphagia requiring tube feeding and later gastrostomy, muscle weakness, and gait ataxia. Clinical examination on admission for investigations revealed absent speech and ambulation without purposeful arm or leg movements, severe axial hypotonus with head lag, and hypertonus of the extremities associated with dystonic movements. Muscle tendon reflexes were normal while the Babinski sign was positive. There were no clinical signs of peripheral neuropathy. Ophthalmologic examination showed nystagmus. Routine laboratory investigations including serum creatine kinase were normal except for hyperlactatemia with a maximum blood lactate level of 6.75 mmol/L (reference value 0.5–1.7 mmol/L). CSF analysis showed an increased lactate level up to a maximum of 3.6 mmol/L (reference value 0.5–1.8 mmol/L). The CSF albumin level and the CSF/plasma albumin ratio were increased to 2,129 mg/L (reference value <150 mg/L) and 51.7 (reference value <5), respectively, compatible with disruption of the blood-brain barrier. Metabolic investigations showed normal acylcarnitines in serum and normal amino acids in plasma and urine. Organic acids in urine were normal except for an increased lactate level up to a maximum of 130 mmol/mol creatine (reference value <20 mmol/mol creatine). Muscle mitochondrial investigations were performed at age 3 1/12 and 9 years with identical findings of an isolated cytochrome c oxidase deficiency with enzyme activities of 1.38 and 1.36 k/mg protein, respectively (reference value 6.1–15 k/mg protein).

The girl was treated with carnitine, aspartic acid, and coenzyme Q10 but continued to have exacerbations with clinical deterioration associated with infections until age 5 ½ years. The disease has since then stabilized, and she has gradually improved without further exacerbations. The following years, she retained her speech and motor functions, albeit with residual impairment in the form of distal spasticity in the lower extremities, mild ataxia with intention tremor, and mild generalized muscle weakness. There were no clinical signs of peripheral neuropathy. She was operated at age 9 years with percutaneous fractionated Achilles tenotomy and at age 16 years with removal of her gastrostomy. Vision, hearing, and cardiologic examinations have been normal. EEGs have been pathologic with increased amount of low frequency background activity and sparse epileptiform discharges, but she has had no seizures. At last examination at age 25 years, she was 156 cm tall (−2 SD on the Swedish growth chart; target height 165 cm), and her weight was 56 kg (−1 SD). She has followed the normal education program in compulsory school and upper secondary school with limited support. Now she lives independently and cares for her self with minimal parental support. She works part time in a preschool class. A summary of results from the clinical investigations is presented in the table.

**Table:**
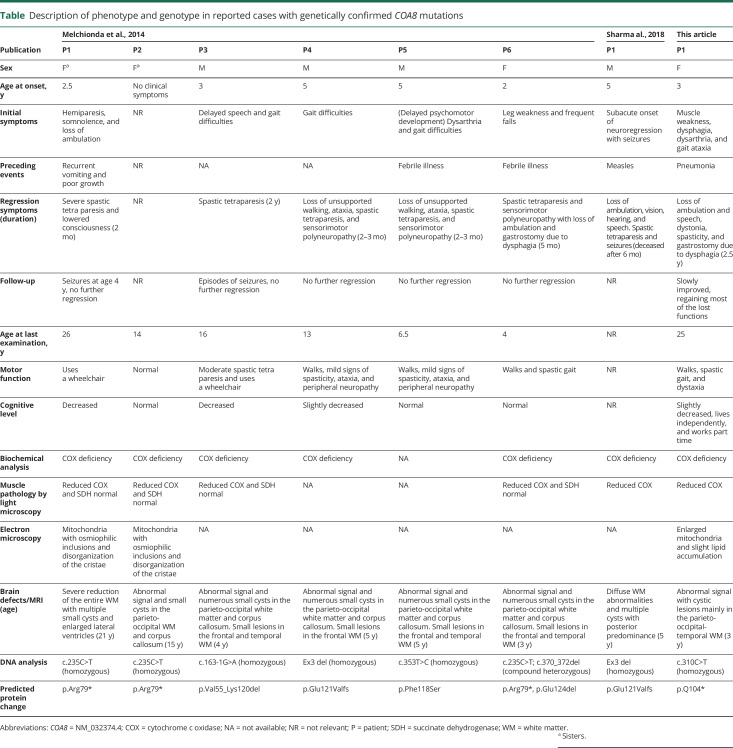
Description of phenotype and genotype in reported cases with genetically confirmed *COA8* mutations

**Figure 1:**
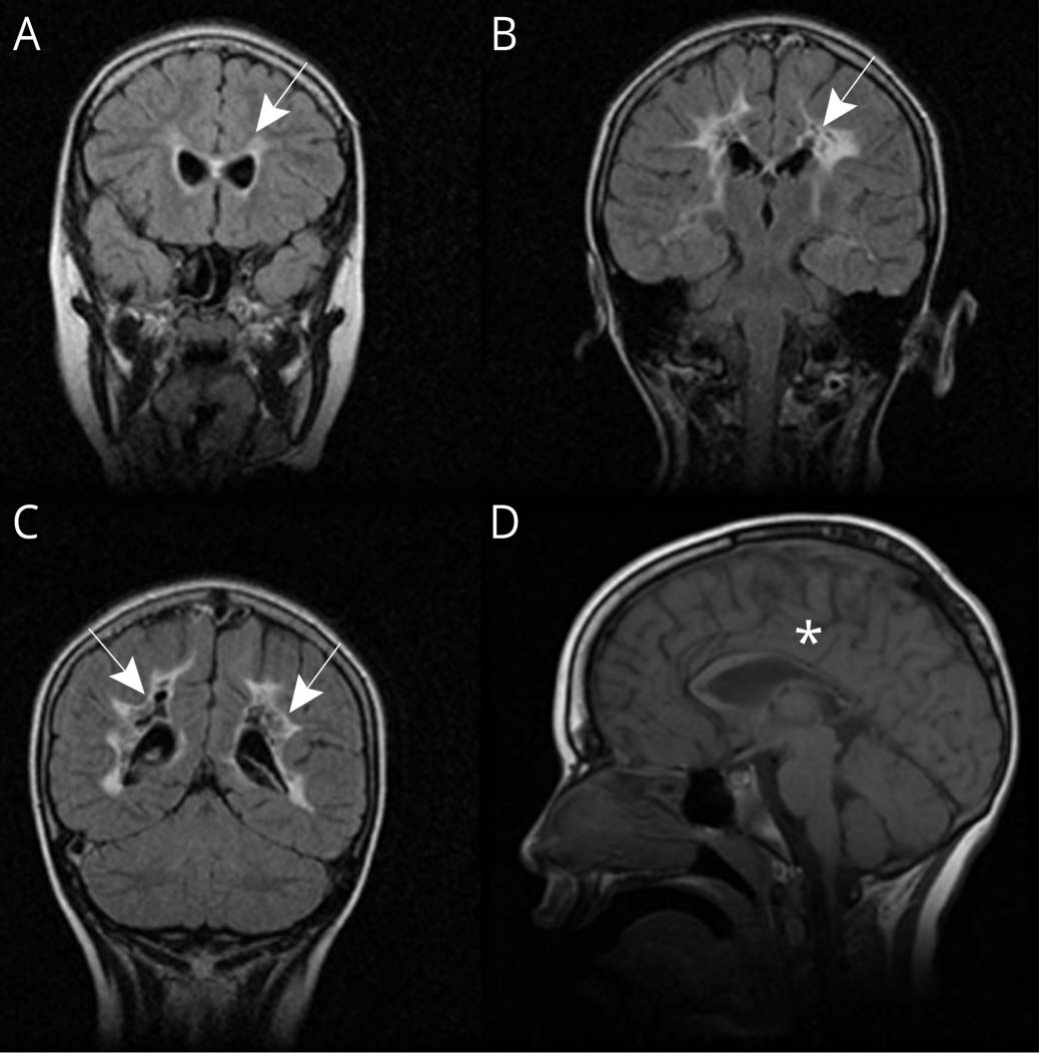
Neuroimaging MRI of the brain at age 8 ½ years with coronal T2-FLAIR FSE (TR/TE = 8,002/161.5 ms; A–C) and sagittal T1 (TR/TE = 440/18 ms; D) showing increased signal with multiple destructive cysts and localized atrophy in supratentorial white matter (white arrows; A–C) and involvement of the corpus callosum with atrophy and localized cysts with decreased signal (white asterisk; D). TE = echo time; TR = repetition time.

MRI of the brain was performed at 3 2/12 years showing bilateral symmetrical cavitating leukoencephalopathy, initially involving the occipital, occipito-parietal, and occipito-temporal regions and to a lesser degree the frontal lobes as well as the genu and splenium of the corpus callosum. The abnormalities were localized to supratentorial white matter and were characterized by high signaling on T2- and low signaling on T1-weighted imaging in addition to multiple destructive cystic changes. Also, streaky areas of contrast enhancement were detected compatible with disruption of the blood-brain barrier. Follow-up examination at age 4 11/12 years showed more involvement of the corpus callosum and frontal lobes and also involvement of the U-fibers as well as more widespread disruption of the blood-brain barrier. MRI of the brain at age 8 ½ years (figure 1, A–D) showed somewhat less pronounced supratentorial white matter changes and the development of localized white matter atrophy. MRI spectroscopy on this occasion did not show any lactate elevation. There was no involvement of the basal ganglia, brain stem, or cerebellum.

### Morphologic analysis

**Figure 2:**
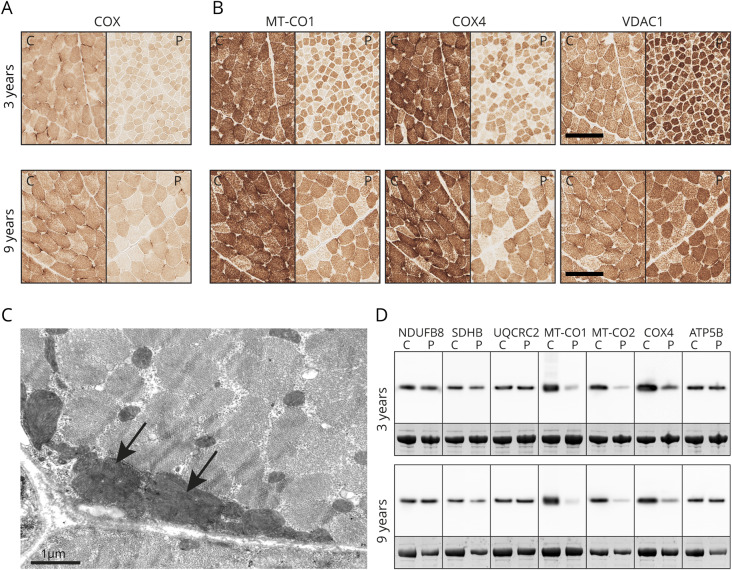
Morphologic and Western blot analysis Serial sections from muscle biopsies of the patient at age 3 and 9 years and control specimens. (A) Enzyme histochemistry showed reduced COX activity in the patient. (B) Immunohistochemistry showed lower expression levels of complex IV subunits MT-CO1 (ab14705) and COX4 (ab110261). VDAC1 (ab14734) was used as a mitochondrial marker. Scale bar is 100 μm. (C) Electron microscopy studies showed the presence of enlarged mitochondria (arrows). (D) Western blot analysis using antibodies for subunits of the respiratory complexes I-V as indicated by gene name (NDUFB8; ab110242, SDHB; ab14714, UQCRC2; ab14745, MT-CO2; ab110258, ATP5B; ab14730). Note the reduced expression level of complex IV subunits MT-CO1, MT-CO2, and COX4 (same antibodies as for IHC). All antibodies were purchased from Abcam. The band corresponding to myosin heavy chain (MyHC) was used as loading control (the lower band in each panel). C = control; P = patient.

Muscle biopsy from left vastus lateralis at age 3 years demonstrated normal fiber caliber variation, no internalized nuclei, no ragged red fibers, a slight increase in subsarcolemmal mitochondria, and a slight increase of lipid droplets. Enzyme histochemistry revealed COX deficiency, and immunohistochemistry revealed loss of complex IV subunits, MT-CO1, MT-CO2 (data not shown), and COX4, respectively (figure 2, A and B). Subunits of complexes I‒III and V (NDUFB8, SDHB, UQCRC2, and ATP5B) showed slightly increased expression compatible with the increased mitochondrial mass, as demonstrated by the mitochondrial marker VDAC. The muscle biopsy from left vastus lateralis at age 9 years revealed the same alterations as the biopsy at age 3 years, although fiber size had increased (figure 2, A and B). Electron microscopy studies showed the presence of enlarged mitochondria (figure 2C) and slight lipid accumulation.

### Western blot

Western blot analysis of repeat muscle biopsies from the patient at age 3 and 9 years and age-matched controls demonstrated reduced expression level of complex IV subunits MT-CO1, MT-CO2, and COX4 in the patient (figure 2D).

### Molecular genetic analysis

**Figure 3:**
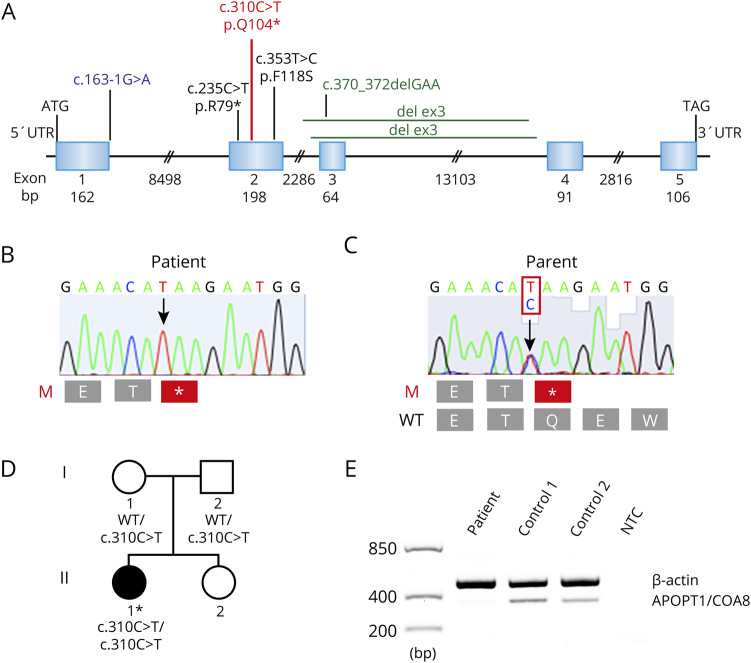
Molecular genetics (A) Illustration showing all identified pathogenic mutations in *COA8*. The novel mutation in this study c.310C>T, p.Q104* is marked in red (NM_032374.4). (B) Chromatogram demonstrating the homozygous c.310T>C mutation in *COA8* in the patient and (C) in one of the heterozygous parents. (D) Pedigree of the family, filled symbol represents affected individual. Asterisk indicates individual analyzed by whole-genome sequencing. (E) The expression patterns of *COA8* and of the *β-actin* gene (NM_001101.3) as an internal control were investigated with reverse transcriptase PCR using complementary DNA derived from messenger RNA extracted from skeletal muscle taken from the patient and control individuals and showed loss of *COA8* transcripts in the patient compared with the controls. NTC = no-template control.

WGS showed that the patient had a homozygous nonsense mutation in *COA8* (c.310C>T; p.(Q104*)) (NM_032374.4) (figure 3B). The mutation was identified in 12 of 251380 alleles, but only in heterozygous individuals in gnomAD. The variant is classified as pathogenic according to American College of Medical Genetics and Genomics guidelines. Other identified variants in candidate genes associated with mitochondrial disorders were predicted to be likely benign or with uncertain significance. Genetic analysis of the parents revealed that they were both heterozygous for the mutation (figure 3, C and D). Expression analysis showed loss of *COA8* transcripts in the patient sample relative to control samples (figure 3E).

## Discussion

We describe the eighth patient known to date with pathogenic mutations in *COA8* (table).^2,3^

The remarkable clinical course with rapid clinical deterioration affecting both cognitive and motor functions over months followed by stabilization and slow improvement over several years appears to be a characteristic finding in this disease since it has been described in some of the 7 previously reported cases (table).^2,3^ This benign course is otherwise unusual in mitochondrial encephalomyopathies with isolated COX deficiency.^1^ In addition, the MRI findings showing cavitating leukoencephalopathy affecting mainly the posterior parts of the white matter and the adjacent corpus callosum and sparing the infratentorial regions seem to be a very characteristic pattern and different from other cavitating leukoencephalopathies (table 1).^2,3,5^

By protein analyses, we identified profound deficiency of subunits MT-CO1, MT-CO2, and COX4, which are integral to the assembly of complex IV. This loss of complex IV subunits in muscle may explain the deficient enzyme activity. The relation between COA8 deficiency and loss of complex IV subunits is not exactly known, but defective assembly of COX was demonstrated in knockout mice and immortalized fibroblast-derived cell lines from *COA8*-mutated patients.^6^ In a *Drosophila* knockdown model of COA8 deficiency, it was demonstrated that dCOA8 protects the flies from oxidative stress in vivo, supporting in vitro results from studies on mammalian cells indicating that COA8 is involved in protection of COX from reactive oxidative species.^7,8^

In conclusion, we report long-term follow-up of a case with *COA8*-associated mitochondrial encephalomyopathy. We demonstrated enzymatic cytochrome c oxidase deficiency and reduced expression of complex IV subunits by protein analyses of muscle tissue, together with ultrastructural abnormalities. Our study provides additional evidence for an association between *COA8* and leukoencephalopathy with complex IV deficiency, typical MRI pattern, and characteristic clinical course.

## Glossary

COX: cytochrome c oxidase
WGS: whole-genome sequencing
